# Impaired stem cell differentiation and somatic cell reprogramming in DIDO3 mutants with altered RNA processing and increased R-loop levels

**DOI:** 10.1038/s41419-021-03906-2

**Published:** 2021-06-21

**Authors:** Agnes Fütterer, Amaia Talavera-Gutiérrez, Tirso Pons, Jesús de Celis, Julio Gutiérrez, Verónica Domínguez Plaza, Carlos Martínez-A

**Affiliations:** 1 Department of Immunology and Oncology, Centro Nacional de Biotecnología (CNB-CSIC), 28049 Madrid, Spain; 2 Transgenesis Unit, CNB & Centro de Biología Molecular Severo Ochoa, CSIC-Universidad Autónoma de Madrid, 28049 Madrid, Spain

**Keywords:** Cell biology, Embryonic stem cells

## Abstract

Embryonic stem cell (ESC) differentiation and somatic cell reprogramming are biological processes governed by antagonistic expression or repression of a largely common set of genes. Accurate regulation of gene expression is thus essential for both processes, and alterations in RNA processing are predicted to negatively affect both. We show that truncation of the *DIDO* gene alters RNA splicing and transcription termination in ESC and mouse embryo fibroblasts (MEF), which affects genes involved in both differentiation and reprogramming. We combined transcriptomic, protein interaction, and cellular studies to identify the underlying molecular mechanism. We found that DIDO3 interacts with the helicase DHX9, which is involved in R-loop processing and transcription termination, and that DIDO3-exon16 deletion increases nuclear R-loop content and causes DNA replication stress. Overall, these defects result in failure of ESC to differentiate and of MEF to be reprogrammed. MEF immortalization restored impaired reprogramming capacity. We conclude that DIDO3 has essential functions in ESC differentiation and somatic cell reprogramming by supporting accurate RNA metabolism, with its exon16-encoded domain playing the main role.

## Introduction

Developmental differentiation and cell reprogramming are related biological processes that share certain common players and mechanisms [1]. Transcription networks are a hallmark, and include the pluripotency-related transcription factors OCT4, KLF4, and SOX2, all involved in self-renewal in pluripotent embryonic stem cells (ESC), as well as c-MYC (OKSM). These factors must be downregulated to permit ESC differentiation, and reexpressed for somatic cell reprogramming [2–4]. These transitions imply modifications in cell structures, cytoskeleton organization, cell polarity, and cell-cell contacts [5], all of which require changes in gene expression profiles.

Coordinated RNA processing and metabolism is a prerequisite for differentiation and reprogramming. Complex mechanisms such as RNA splicing [6, 7], alternative termination [8], stability and transport [9], and expression regulation by miRNAs [10] are necessary. Growing evidence also links R-loop formation and dynamics to the physiological processing of gene regulation [11, 12]. R-loops consist of a DNA/RNA hybrid that leaves the non-template DNA as single strand, a possible source of DNA breaks. Although initially described as transcription byproducts that threaten genome integrity, R-loops are important transcription regulators [11, 13–15] that participate in cell fate determination [16, 17] and somatic cell reprogramming [18]. Deregulation of R-loops leads to cell stress, especially when transcription meets replication [19–21]. RNA-binding proteins and splicing factors prevent R-loop formation, and processing factors such as RNaseH and helicases can dissolve them [22].

The *DIDO* gene participates in several of these processes. It is connected to chromatin remodeling processes and is stemness-related [23]. Its N-terminal truncation (DIDOΔNT) leads to genomic instability [24, 25] and yields live mice, although they develop myeloid neoplasms [26]. In contrast, DIDO3 isoform-specific C-terminal deletion (DIDO3ΔCT) is embryonic lethal in mice; ESC derived from such mutants do not differentiate in vitro, but maintain self-renewal capacity [27]. DIDO3 is implicated in correct isoform splicing. DIDO3ΔCT mutants do not express DIDO1, its smallest isoform [28], which must be upregulated during differentiation [23] and is involved in downregulation of stemness genes [28]. The DIDO3 and splicing factor SFPQ interaction was recently described, and DIDO3 was linked to correct, efficient RNA splicing [29].

Here we describe embryonic lethality of the conditional DIDO3ΔE16 mutant. Its ESC failed to differentiate in vitro, and RNA-seq analysis showed isoform differences and aberrations in RNA termination. Among the genes affected we found *POU5f1*/OCT4, and identified DIDO3 DNA binding at the *POU5f1* 3′UTR region. ChIP-seq analysis indicates a relationship between DIDO3 and RNA pol II binding and R-loops at the 3′ gene ends. This link is strengthened by DIDO3 C terminus interaction with the DHX9 helicase, by co-immunoprecipitation of both proteins with R-loops, and by results from DHX9 knockdown experiments. We found higher R-loop levels in ESC and primary mouse embryonic fibroblasts (MEF) from DIDO3ΔE16 mutants, which result in DNA damage and lead to replication stress; primary MEF thus fails to undergo somatic cell reprogramming.

Based on these data, we propose a model that links ESC differentiation and somatic cell reprogramming with cell stress caused by impaired R-loop regulation, RNA splicing, and termination.

## Results

### Embryonic lethality and ESC differentiation defects following conditional deletion of DIDO exon16

**Fig. 1:**
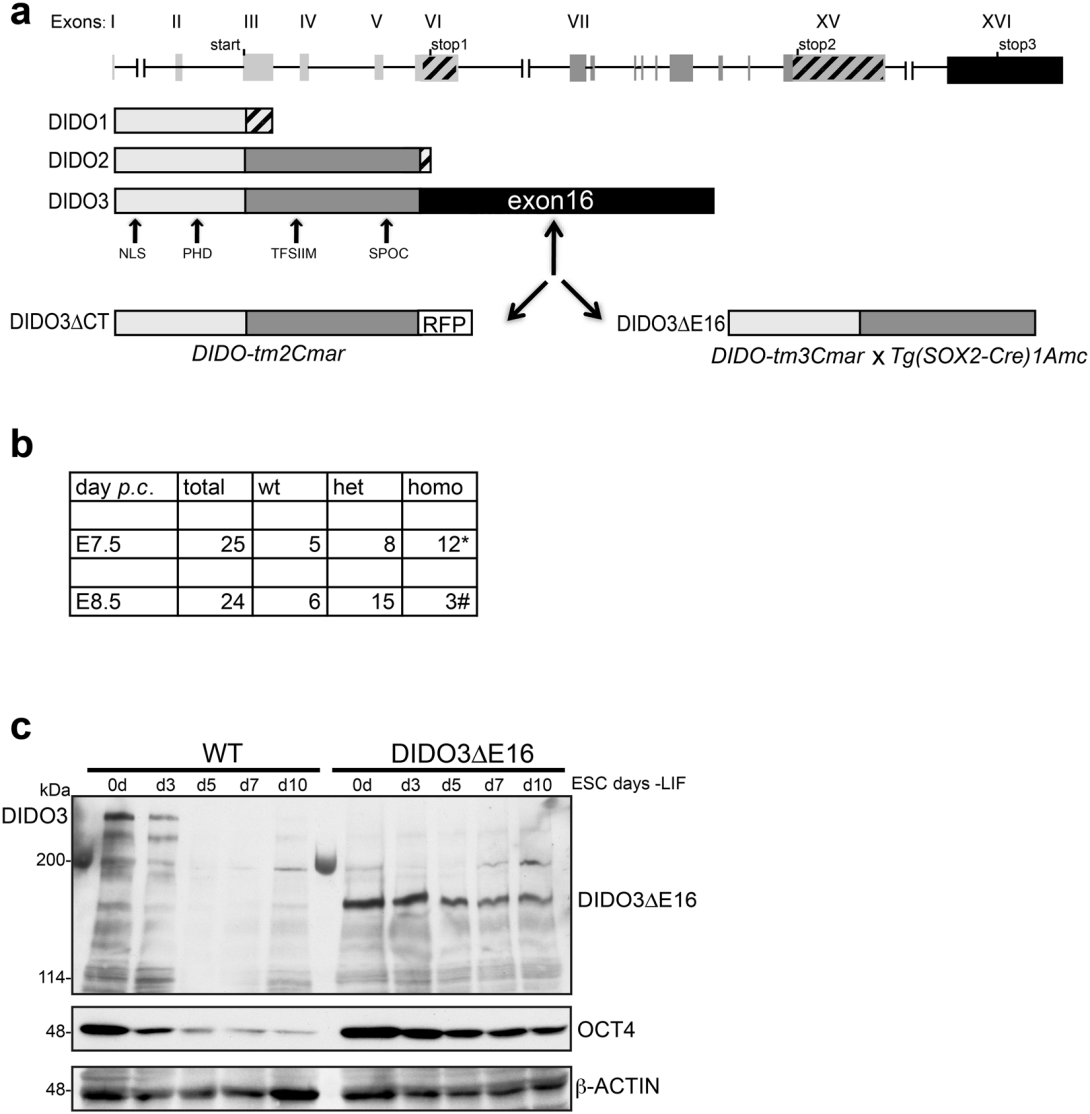
DIDO3ΔE16 mutant and its phenotype in vivo and in vitro. **a** *DIDO* gene structure and DIDO protein isoforms and their domains in WT, constitutive DIDO3ΔCT, and conditional DIDO3ΔE16 mutants; NLS nuclear location signal, PHD plant homeodomain, TFSIIM transcription elongation factor S-II subunitM, SPOC Spen paralog and ortholog module, RFP red fluorescent protein. **b** Embryonic lethality at E7.5 and E8.5 post-coitum of DIDO3ΔE16 homozygous (homo) embryos compared to wild type (WT) and heterozygous (het) embryos. The asterisk (*) indicates morphological anomalies and the octothorpe (#), signs of resorption. **c** Western blot analysis of cell lysates from embryonic stem cells (ESC) and embryonic bodies at different times (d3, d5, d7, d10) after leukemia inhibitory factor (LIF) withdrawal from WT and DIDO3ΔE16, tested by monitoring DIDO3 or DIDO3ΔE16 and OCT4 protein levels, using β-ACTIN as loading control.

We crossed *DIDO-tm3Cmar* mice bearing the floxed DIDO3-specific exon16 with *Tg(SOX2-Cre)1Amc* mice [30] for Cre-mediated deletion (Fig. 1a). After intercrossing heterozygous DIDO3ΔE16 mice, we examined F2 embryos; there were no live embryos with homozygous deletion beyond d7.5 (Fig. 1b). Established homozygous ESC showed impaired differentiation in vitro, seen as persistence of OCT4 and DIDO3ΔE16 after LIF (leukemia inhibitory factor) withdrawal (Fig. 1c), as described for constitutive deletion [27, 28]. Here we refer to deletion of the floxed mutant as DIDO3ΔE16 and to the previous C-terminal deletion mutant as DIDO3ΔCT; both affect the same exon16.

### DIDO3 is implicated in alternative isoform splicing and RNA transcription termination

We isolated RNA from WT, DIDO3ΔE16, and from DIDO3ΔCT ESC reconstituted with HADIDO3, and performed stranded RNA-sequencing (RNA-seq). We aligned sequences to the mouse reference genome (GRCm38/mm10 assembly) and analyzed data with the DEXSeq [31] program, which evaluates differential usage of exonic regions as a proxy for alternative isoform regulation. We found 290 exons with significant differences in WT compared to DIDO3ΔE16 ESC, and almost all exons recovered normal expression in DIDO3ΔCT ESC after HADIDO3 reconstitution (Supplementary Table 1; Supplementary Fig. 1a). As predicted, *DIDO* was among the genes found and RNA-seq data confirmed the reported lack of splicing to the DIDO1 isoform [23, 28]; HADIDO3 reconstitution restored DIDO1 expression (Supplementary Fig. 1b).

For observed 3′UTR read-throughs of transcripts, we reanalyzed the RNA-seq data and calculated expression differences with 2 kb added downstream of the defined 3′UTRs. Genes whose expression of prolonged 3′UTR in DIDO3ΔE16 recovered normal levels after reconstitution with HADIDO3 are shown (Supplementary Table 2).

**Fig. 2:**
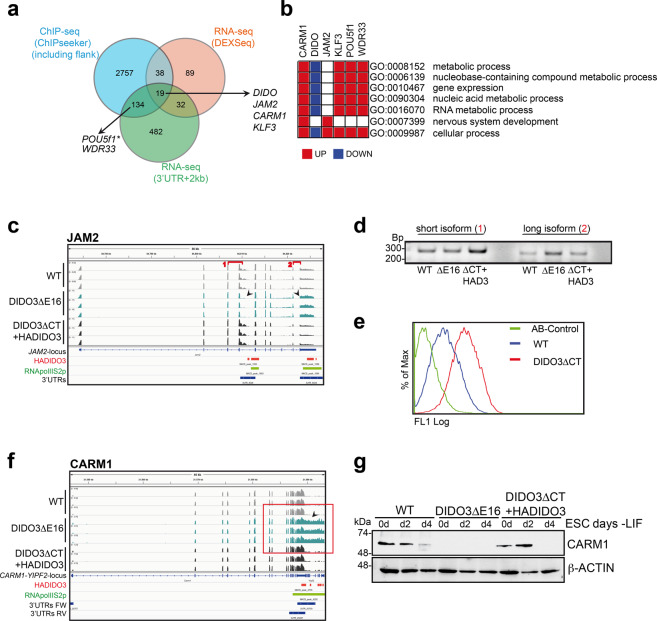
DIDO3ΔE16 ESC expression alterations detected by RNA-seq, with effects on protein expression. **a** The Venn diagram depicts the relationship of genes identified for altered expression in DIDO3ΔE16 ESC compared to WT, and restored in DIDO3ΔCT+HADIDO3 in RNA-seq (DEXSeq, Supplementary Table 1; 3′UTR + 2 kb, Supplementary Table 2), and a DIDO3 DNA-binding site determined by ChIPseeker of previous ChIP-seq data (Supplementary Table 3); some relevant examples are highlighted. Note: (*) Significant alteration (1.5-fold difference; FDR < 0.05) was not detected by RNA-seq data analysis, but RT-PCR confirmed a prolonged 3′UTR of the transcript only in the DIDO3ΔE16 mutant ESC. For details, see Results. **b** GO term enrichment analysis and heatmap of the selected genes. UP- and DOWN regulated genes in DIDO3ΔE16 versus WT are shown in red and blue, respectively. Significantly enriched GO terms (biological processes) were identified using DAVID (https://david.ncifcrf.gov/), g: Profiler (https://biit.cs.ut.ee/gprofiler/gost), and GSEA (https://www.gsea-msigdb.org/). For additional details of the enrichment analysis, see Supplementary Fig. 1d. **c** *JAM2* example gene; IGV image shows the *JAM2* locus, aligned and normalized RNA-seq reads of triplicates of WT, DIDO3ΔE16, and DIDO3ΔCT+HADIDO3 ESC as well as DIDO3 ChIP-seq peaks (red) with overlapping RNA pol II S2p peaks (green) and defined 3′UTR regions (blue). **d** Different abundance of *JAM2* short isoform1 and long isoform2 (indicated in c), confirmed in WT, DIDO3ΔE16, and DIDO3Δ+HADIDO3 RNA as tested by RT-PCR. **e** The histogram shows abundance of the JAM2 protein in WT and DIDO3ΔCT ESC as measured by flow cytometry analysis after anti-JAM2 antibody staining. **f** *CARM1* example gene; parameters as in (**b**) for the *CARM1* locus, focusing on 3′UTR read-throughs in the DIDO3ΔE16 mutant (red box). **g** Western blot analysis of lysates of ESC and embryonic bodies without LIF at d2 and d4, comparing CARM1 protein levels in WT, DIDO3ΔE16, and DIDO3ΔCT+HADIDO3 cells; β-ACTIN used as loading control.

As DIDO3 binds to chromatin [28], we tested whether expression alterations are linked to DIDO3-DNA binding, using data from ChIP-seq experiments ([28] GEO: GSE85029). HADIDO3 peaks were found preferentially in genome regions with a high density of gene coding sequences (Supplementary Fig. 1c). We associated HADIDO3 binding sites to the gene with the nearest transcription start site (TSS) and to flanking genes (up to 5 kb) and reanalyzed the ChIP-seq peaks with the ChIPseeker program [32], which identified peak location (Supplementary Table 3). Combined analysis of ChIP- and RNA-seq data showed candidate genes with expression alterations in DIDO3ΔE16 ESC when recovered by HADIDO3 expression (Fig. 2a). Gene ontology analysis of affected genes showed significantly enriched biological processes (Supplementary Fig. 1d); we depicted the expression pattern of selected genes in these processes (Fig. 2b).

To validate the results, we used RT-PCR and/or western blot to correlate changes in RNA and protein expression. We selected *KLF3*, involved in proliferation, development, and differentiation [33], and *WDR33*, identified by its role in 3′RNA processing [34, 35]. For both, we confirmed alterations in RNA expression; DIDO3ΔE16 ESC preferentially express the longer *KLF3* isoform (Supplementary Fig. 2a, b) and a longer 3′UTR RNA region of *WDR33* (Supplementary Fig. 2d, e). Analysis with available antibodies detected no alterations in protein expression in either case (Supplementary Fig. 2c, f, respectively).

Additional candidates were *JAM2*, expressed in ESC [36] and implicated in cell polarization [37], as is DIDO [27, 28], and *CARM1*, identified in early developmental differentiation [38]. RNA expression of the large *JAM2* isoform was increased (Fig. 2c, d), and surface protein expression in mutant was elevated ESC (Fig. 2e). For *CARM1*, we observed a RNA read-through at its termination region (Fig. 2f); this resulted in an almost complete absence of CARM1 protein in DIDO3ΔE16 ESC, which was restored following HADIDO3 expression (Fig. 2g).

### *POU5f1* transcriptional termination and regulation by DIDO

**Fig. 3:**
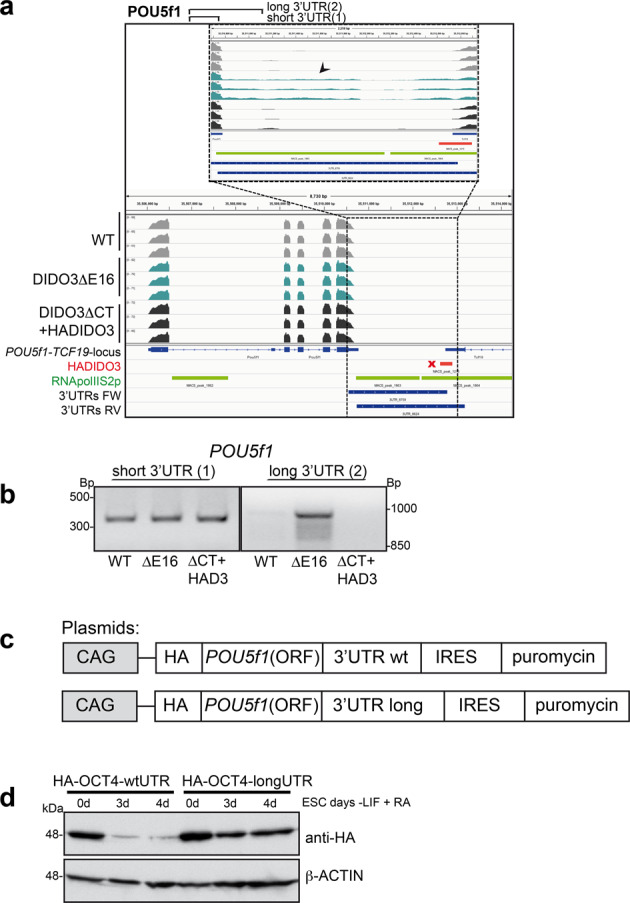
DIDO transcriptional termination and regulation of *POU5f1* RNA. **a** *POU5f1* example gene: IGV image of the *POU5f1* locus with parameters as in Fig. 2c, showing 3′UTR read-through in the DIDO3ΔE16 mutant. **b** Different abundance of *POU5f1* short and long 3′UTR (indicated in **a**) confirmed in WT, DIDO3ΔE16, and DIDO3ΔCT+HADIDO3 RNA as tested by RT-PCR. **c** Scheme of expression plasmids: introducing 5′ HA-tag sequences and short or long 3′UTR to the open reading frame (ORF) of *POU5f1* DNA. **d** Western blot analysis of lysates from ESC and embryonic bodies at d3 and d4 after LIF withdrawal plus retinoic acid from WT ESC transfected with (plasmids as in **c**), developed with anti-HA antibody; β-ACTIN was used as loading control.

The *POU5f1* gene encodes OCT4, the main core component for ESC self-renewal [39–41]. ESC from DIDO3ΔCT [27] and DIDO3ΔE16 have an OCT4 downregulation delay during in vitro differentiation. ChIP-seq data identified a HADIDO3 binding site of the *POU5f1* 3′UTR within the 2-kb regulatory region (Fig. 3a). We confirmed DIDO3 binding by quantitative PCR and found 2.5 ± 1.2-fold ChIP enrichment when normalized to input levels. RT-PCR verified a read-through at transcription termination in the DIDO3ΔE16 ESC (Fig. 3b). To study the potential influence of *POU5f1* 3′RNA extension on OCT4 protein persistence in DIDO3ΔE16 ESC, we constructed two *POU5f1* expression plasmids, one with WT 3′UTR and one with the extended 3′UTR, both HA-tagged on the 5′ site to distinguish their recombinant HA-OCT4 products from endogenous OCT4 (Fig. 3c). After stable transfection into WT ESC, clones with similar expression levels were seeded without LIF and stimulated with retinoic acid to induce differentiation and OCT4 downregulation. Western blot analysis showed prolonged expression of HA-OCT4 bearing the long 3′UTR versus the WT 3′UTR (Fig. 3d).

### DIDO3 binds mainly in 3′ gene regions to chromatin, overlapping with RNA polymerase II, and associates with DHX9 and R-loops

**Fig. 4:**
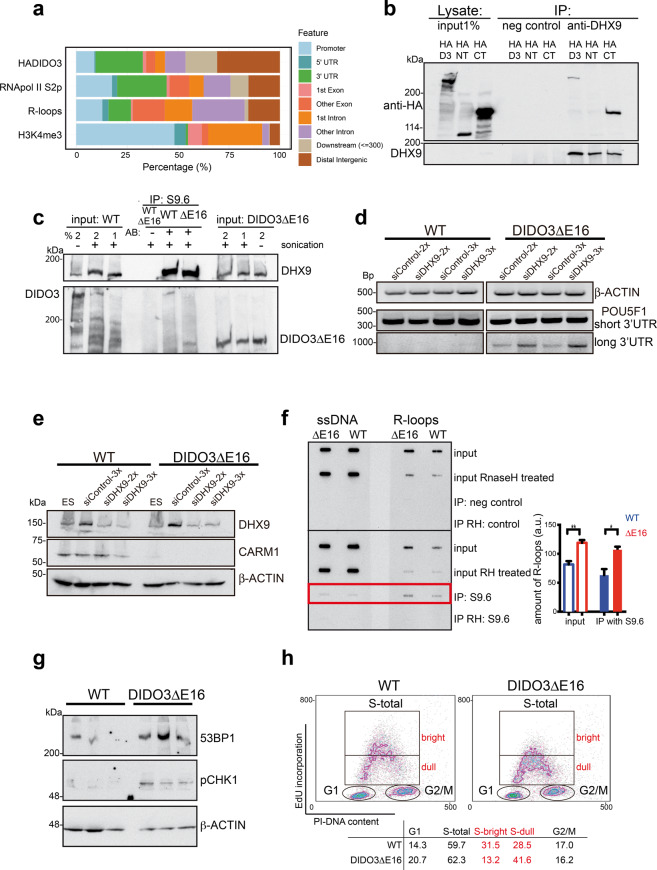
Coexistence of DIDO3, RNA pol II, DHX9, and R-loops, and alterations in DIDO3ΔE16 mutant ESC. **a** BED peak files were imported to RStudio and annotated using the R/Bioconductor package ChIPseeker. Peak annotations are depicted as a percentage for HADIDO3, RNA pol II S2p, R-loops, and H3K4me3 (histone 3 trimethylated on lysine 4). **b** Co-immunoprecipitation of HA-tagged DIDO3, -common N-terminal region, or -DIDO3-exon16-specific C-terminal part (for constructs see [28]), and DHX9 helicase. **c** Co-immunoprecipitation of DIDO3 or DIDO3ΔE16 proteins and DHX9 helicase at R-loops precipitated with the S9.6 mAb, from sonicated chromatin of WT or DIDO3ΔE16 ESC. **d** Abundance of *POU5f1* short and long 3′UTR after two or three applications of siDHX9 or siControl in WT and DIDO3ΔE16 ESC; β-ACTIN was used as control for equal RNA amounts. **e** Western blot analysis of DHX9 and CARM1 expression in WT and DIDO3ΔE16 ESC after two or three applications of siDHX9 or siControl; β-ACTIN was used as loading control. **f** Left: representative example of slot-blot analysis of restriction enzyme-digested genomic DNA, before and after immunoprecipitation with S9.6 mAb, alone or treated with RNaseH1 from WT or DIDO3ΔE16 ESC. Probes were used in duplicate; the first was developed with anti-ssDNA mAb and the second with anti-R-loop mAb S9.6. Right: quantification of slot–blot bands normalized to ssDNA values with ImageJ. Bars represent mean ± SEM. Statistical analysis was done with two-tailed Student’s *t*-test, **P ≤ 0.01; *P ≤ 0.05; DIDO3ΔE16, *n* = 3; WT, *n* = 3. **g** Western blot analysis of total lysates from WT and DIDO3ΔE16 ESC developed with anti-53BP1 and anti-phosphorylated CHK1 (pCHK1) antibodies; β-ACTIN was used as loading control. **h** Contour density plot of WT and DIDO3ΔE16 ESC after a 30-min EdU pulse, showing EdU incorporation versus DNA content. The percentages of events in cytometric gates defining different phases of the cell cycle are depicted.

DIDO3 interacts with RNA pol II [28]. Intersection analysis of HADIDO3 ChIP-seq peaks (GEO: GSE85029) with RNA pol II peaks (GEO: GSE34520 [42]) showed a DIDO3 overlap with RNA pol II, especially with pS2 (phosphorylated at serine 2), of ~66% (Fig. 4a; Supplementary Table 4), which suggests cooperation in regulation [43]. RNA pol II pS2 is associated with transcription termination [44–46], as are R-loop dynamics [12]. High R-loop levels at the 3′ end of genes suggest an R-loop role in preventing transcriptional read-through into adjacent genes [47]. Including R-loop ChIP-seq peaks in ESC (GEO: GSE70189 [47]) to upper intersections (Supplementary Table 4) yielded ~45% of DIDO3 peaks with simultaneous RNA pol II S2p and R-loop peaks (Supplementary Fig. 3a). More than 50% of these genes had these peaks at the 3′UTR or within 3 kb downstream.

Genes related to RNA splicing, termination, elongation rate, and R-loop dynamics were identified by ChIP-seq and/or RNA-seq (Supplementary Table 5). We concentrated on DHX9, a helicase involved in R-loop suppression and transcription termination [48], that interacts with DIDO3 in MEF [29]. We performed co-immunoprecipitation experiments using anti-DHX9 antibody on lysates of ESC overexpressing HADIDO3, or the HA-N- or HA-C-terminal part of DIDO3, developed the western blot with anti-HA antibody, and found co-immunoprecipitation with whole DIDO3 and its C terminus, but not with the N terminus (Fig. 4b).

DHX9 is present in the DNA/RNA hybrid interactome [48]. We tested for DIDO3 at genomic sites enriched in these hybrids, using the S9.6 mAb to detect R-loops, as it recognizes DNA/RNA hybrids. We refer to its signal as R-loops (albeit aware that not necessarily all stable DNA/RNA hybrids in the genome are limited to R-loops). Sonicated chromatin of nuclear extracts from WT and DIDO3ΔE16 ESC was immunoprecipitated with mAb S9.6. Western blot was developed with anti-DIDO and -DHX9 antibodies, and both co-immunoprecipitated with R-loops (Fig. 4c). We reduced DHX9 protein levels using siRNA and tested for DIDO3-sensitive transcription termination by RT-PCR, focusing on the prolonged 3′UTR expression of *POU5F1* as a read-through indicator. We found further increases in read-through events in siDHX9-treated mutant compared to control siRNA-treated ESC. A faint band was now detected in siDHX9-treated WT ESC, indicating read-through events (Fig. 4d). No influence was observed on the abolished CARM1 levels in mutant ESC, but treated WT ESC now also showed reduced CARM1 expression (Fig. 4e). Both results suggested that DHX9 function is necessary for correct transcription termination.

### DIDO3ΔE16 ESC have more R-loops and show signs of replication stress

We measured R-loop levels in genomic DNA from WT and DIDO3ΔE16 cells in slot–blot experiments before and after immunoprecipitation with the S9.6 mAb. S9.6 immunoreactivity was significantly higher in mutant versus WT samples (Fig. 4f), suggesting altered R-loop dynamics in mutant cells. Control DIDO3ΔE16 and WT genomic DNA treated with RNases of different substrate preference showed signal reduction only for RNaseH1 (Supplementary Fig. 3b). qPCR of the DRIP (DNA/RNA IP) probes showed abundant amplification of *CARM1* and *POU5f1* 3′ gene regions, confirming R-loop presence in these regions. *SOD3*, a gene not expressed in ESC with no R-loops, was the negative control (Supplementary Fig. 3c).

To test the influence of ectopic expression of mouse RNaseH1 on R-loops in DIDO3-dependent transcription termination, we used two distinct HA-tagged RNaseH1 plasmids in transient or stable transfections. We detected protein expression with anti-HA antibody in western blot (Supplementary Fig. 3d), but no alterations in transcription termination as determined by RT-PCR (Supplementary Fig. 3e).

As increased R-loop levels can provoke genomic instability through DNA breaks and conflicts with the replication machinery, we tested for cell stress-associated proteins [20, 21] such as ATR/CHK1 signaling by CHK1 phosphorylation (pCHK1) [49, 50], or proteins indicative of stress such as 53BP1 [51]. We found pCHK1 only in DIDO3ΔE16 cell lysates, with larger amounts of 53BP1 than in WT lysates (Fig. 4g), indicating cell stress in the mutant ESC. As replication stress leads ultimately to impaired DNA synthesis, we tested EdU (5-ethynyl-2’deoxyuridine) incorporation in asynchronized ESC. Although percentages of WT and mutant ESC in S-phase were comparable, mutant ESC incorporated lower EdU levels (Fig. 4h). Serum-starved mutant ESC enriched in G1-phase similarly maintained lower EdU levels and showed delayed S-phase re-entry compared to WT ESC (Supplementary Fig. 3f).

### DIDO3 C- but not N-terminal deletion leads to higher R-loop levels and replication stress in MEF

We obtained d13.5 MEF from mice heterozygous for the floxed and deleted allele; they were infected with Ad5CMVCre-virus (AdCre) to achieve homozygous deletion of exon16, confirmed by PCR and western blot.

**Fig. 5:**
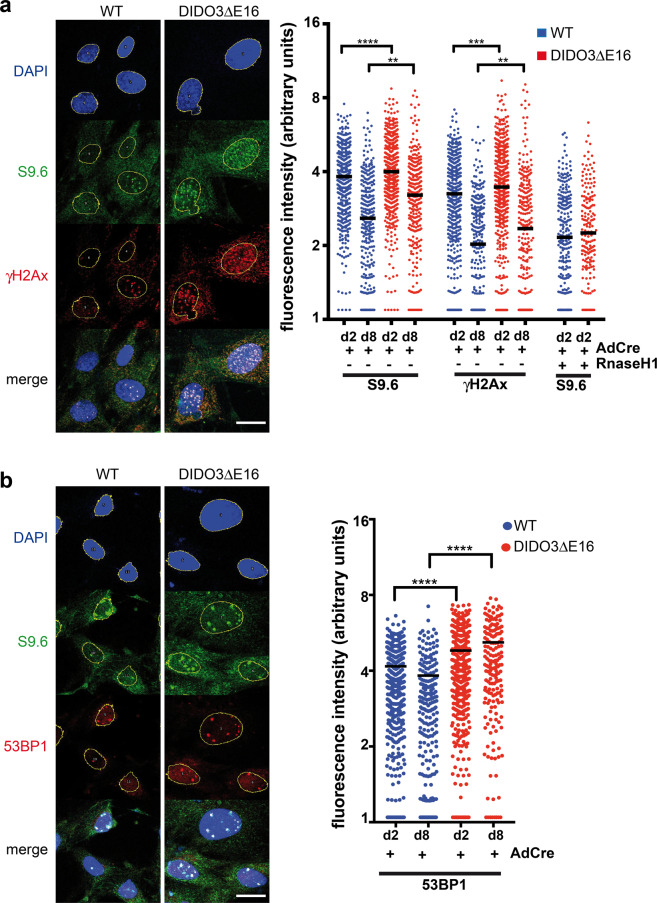
R-loop levels and replication stress in DIDO3ΔE16 MEF. **a** Left: Representative images of WT and DIDO3ΔE16 MEF immunofluorescence after AdCre infection, using DAPI to stain nucleus (blue), S9.6 mAb to stain R-loops (green) and DNA damage-indicating antibody γ-H2Ax (red). Yellow circles depict nucleus corresponding to DAPI-positive staining and as region of interest (ROI) for ImageJ quantification of fluorescence intensity. Bar = 25 μm. Right: fluorescence intensity of S9.6 mAb for R-loops and γ-H2Ax for DNA damage was quantified in the nuclear region of WT and DIDO3ΔE16 MEF after AdCre infection at indicated times. At least 170 cells were analyzed from three different experiments. Specificity for S9.6 mAb was controlled after RNaseH1 treatment. Scatter dot plot with median is shown. Statistical analysis was performed with the one-way ANOVA test, ****P < 0.0001; ***P < 0.001; **P ≤ 0.01. **b** Left: representative images of WT and DIDO3ΔE16 MEF immunofluorescence after AdCre infection, with DAPI stain to indicate nucleus (blue), S9.6 mAb for R-loops (green), and replication stress-indicating antibody 53BP1 (red). Yellow circles depict the nucleus corresponding to DAPI-positive staining and as region of interest (ROI) for ImageJ quantification of fluorescence intensity. Bar = 25 μm. Right: quantification of 53BP1 fluorescence intensity to detect replication stress in the nucleus of WT AdCre versus DIDO3ΔE16 AdCre at d2 and d8. At least 170 cells were analyzed from three different experiments. Scatter dot plot with median is shown. Statistical analysis was performed with the one-way ANOVA test, ****P < 0.0001.

DIDO3ΔΕ16 and WT primary MEF were labeled with the S9.6 mAb to visualize R-loops, and with γH2Ax antibody to detect DNA damage. As AdCre infection already increased R-loop and γH2Ax levels in WT MEF, we quantified R-loops and DNA breaks in both MEF types after AdCre infection. We labeled MEF at d2 and d8 post infection. Compared to WT, DIDO3ΔΕ16 MEF showed persistent, increased R-loop and γH2Ax levels at both times (Fig. 5a). We used anti-53BP1 to detect replication stress as 53BP1-positive nuclear bodies (NB) [51] and found increased 53BP1-positive NB in DIDO3ΔΕ16 compared to WT MEF (Fig. 5b); replication stress was increased at d8 in mutant MEF.

We also examined DIDOΔNT MEF in immunofluorescence experiments as above with S9.6, γH2Ax, and 53BP1 antibody staining, and found opposing results in DIDOΔNT and DIDO3ΔE16 compared to WT MEF. R-loop levels in DIDOΔNT were significantly lower than in WT MEF (Supplementary Fig. 4a), with γH2Ax (Supplementary Fig. 4a) and 53BP1 levels similar to WT (Supplementary Fig. 4b).

### DIDO3-exon16 is necessary for somatic cell reprogramming

**Fig. 6:**
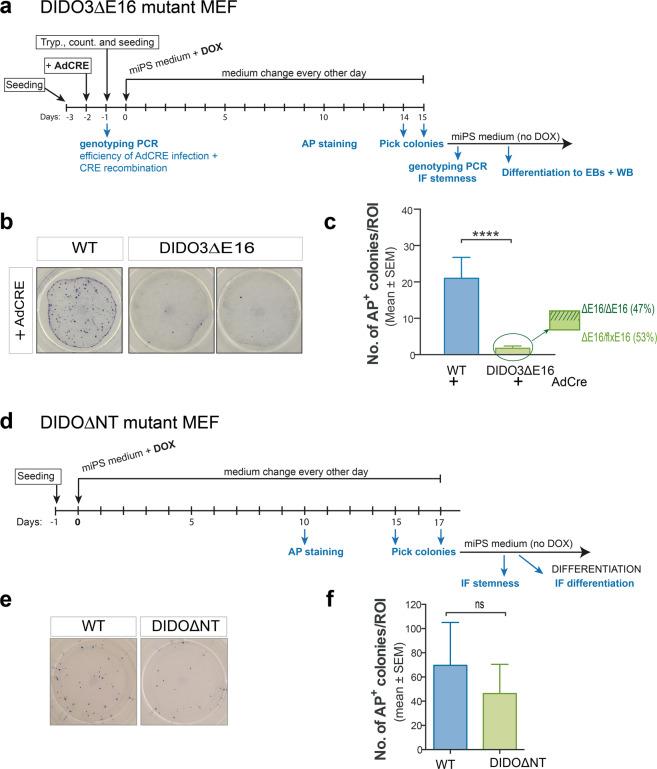
Somatic reprogramming requires DIDO3-exon16 but not the N-terminal region to induce pluripotent stem cells. **a** Scheme for experimental onset and reprogramming procedures for DIDO3ΔE16/4F-MEF. Tryp trypsinization, DOX doxycycline addition, AP alkaline phosphatase staining. **b** Image of representative wells after staining for AP-positive colonies as a pluripotency marker following reprogramming of WT and DIDO3ΔE16 MEF after AdCre infection. **c** Quantification of reprogramming ability of DIDO3ΔE16/4F-MEF versus WT/4F-MEF after AdCre infection, as determined by AP-positive colonies. DIDO3ΔE16, *n* = 8; WT *n* = 8; bars indicate mean ± SEM. Statistical analysis was performed with two-tailed Student’s *t*-test, ****P < 0.0001. Percentage of genotypes in iPS homozygous for *DIDO3ΔE16/DIDO3ΔE16* or heterozygous for *DIDO3ΔE16/DIDO3floxedE16* was determined by PCR. **d** Scheme of experimental onset and DIDOΔNT/4F-MEF reprogramming procedures. **e** Staining for AP-positive colonies as a pluripotency marker after WT and DIDOΔNT MEF reprogramming. **f** Quantification as in (**c**) for DIDOΔNT/4F-MEF (*n* = 3) and WT/4F-MEF (*n* = 3); bars represent mean ± SEM. Statistical analysis was performed with two-tailed Student’s *t*-test and showed no significant differences (ns).

To determine the influence on somatic reprogramming, we crossed DIDO3-exon16 floxed mice to transgenic mice with doxycycline-inducible expression of the reprogramming genes *Tg(tetO-POU5f1,SOX2,KLF4,MYC)1Srn* [52]. Day 13.5 MEF were AdCre-infected for homozygous deletion of exon16, and doxycycline-treated to induce expression of the four circuit stem cell factors necessary for somatic reprogramming [3] (Fig. 6a). PCR and western blot confirmed deletion efficiency before induction (Supplementary Fig. 5a, b). We obtained iPSC from WT MEF, but DIDO3ΔE16 MEF showed severely reduced reprogramming efficiency. Although some colonies stained for the early pluripotency marker alkaline phosphatase (AP) (Fig. 6b), colonies tested for DIDO3 expression showed that half had escaped AdCre deletion and still bore the floxed *DIDO3* allele. In eight independently established DIDO3ΔE16 MEF samples, reprogramming was almost abolished (Fig. 6c).

Like DIDO3ΔE16 ESC, the few iPSC generated from mutant MEF had a correct pluripotency network (Supplementary Fig. 5c), but did not downregulate OCT4 at differentiation onset (Supplementary Fig. 5d).

DIDO3ΔE16 mutants differ from the DIDOΔNT mutants which, despite cell cycle and genomic stability problems [24, 25] and splicing defects [29], show neither embryonic lethality [26] nor ESC differentiation defects. For somatic reprogramming, we tested doxycycline-treated MEF from DIDOΔNT crossed to reprogrammable OKSM mice, and obtained fully reprogrammed iPSC (Fig. 6d, e, f). Like DIDOΔNT ESC, established iPSC were bona fide iPSC with completely normal stemness characteristics (Supplementary Fig. 6a) and able to differentiate into cells of all three germ layers (Supplementary Fig. 6b).

### Immortalization of DIDO3ΔE16 MEF rescues somatic cell reprogramming capacity

**Fig. 7:**
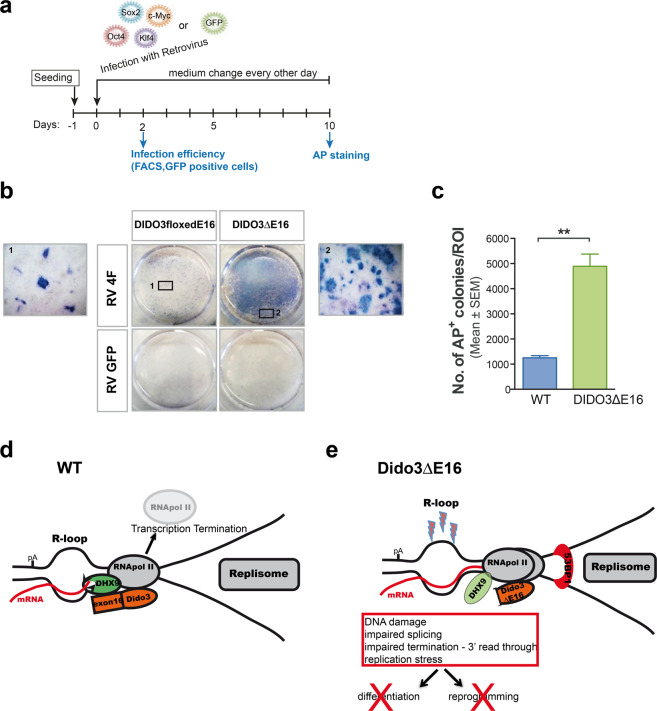
Recovery of reprogramming capacity in immortalized DIDO3ΔE16 MEF, and model for transcription termination in WT and DIDO3ΔE16 cells. **a** Scheme for experimental onset and procedures for reprogramming immortalized DIDO3ΔE16. **b** Top: images of representative wells of AP-positive colonies as a pluripotency marker after infection with OKSM retroviruses, from reprogrammed parental DIDO3floxedE16 before Cre deletion and from DIDO3ΔE16 MEF. Bottom: control wells with GFP-retrovirus infection. **c** Quantification to determine AP-positive colonies; bars represent mean ± SEM. Statistical analysis using two-tailed Student’s *t*-test showed significant differences **P ≤ 0.01. **d** In WT cells, DHX9 binds to RNA pol II and DIDO3 at termination sites, RNA pol II terminates RNA transcription, DHX9 resolves R-loops, and RNA pol II dissociates from DNA. **e** In DIDO3ΔE16 cells, DHX9 function is impaired, R-loops persist, RNA pol II continues reading, DNA damage occurs on the single-stranded DNA, and transcription–replication conflicts provoke cell stress. All these effects abolish differentiation and reprogramming.

We tested whether immortalizing primary MEF by E6/E7 transfection could rescue impaired reprogramming in DIDO3ΔE16 MEF. Immortalized WT and DIDO3ΔE16 MEF [29] were infected with a mixture of the four retroviruses expressing each of the OKSM transcription factors to induce reprogramming, or with GFP-expressing retrovirus as a negative control for reprogramming and positive control for infection efficiency (Fig. 7a). In all experiments, infection efficiency measured by FACS was >80% in both genotypes. After 10 days, AP staining experiments and quantification of reprogramming efficiency (Fig. 7b) showed restored DIDO3ΔE16 reprogramming, with a significant increase compared to immortalized WT MEF (Fig. 7c).

Our results support a model in which DIDO3 interacts and collaborates with DHX9 to regulate R-loops at RNA pol II transcription termination sites (TTS) (Fig. 7d). DIDO3-exon16 deletion leads to altered DHX9 function, induces defects in RNA termination seen as read-throughs, and results in more stable R-loops. All these factors contribute to genomic instability, DNA damage, and replication stress, which in turn abolish stem cell differentiation and somatic cell reprogramming (Fig. 7e).

## Discussion

We previously identified the *DIDO* locus that participates in early embryonic development and ESC differentiation; here we postulate an underlying molecular mechanism and an additional DIDO role in somatic cell reprogramming.

The *DIDO* gene is stemness-related, and C-terminal deletion leads to embryonic lethality and differentiation defects [27, 28]; in contrast, mice with DIDO N-terminal truncation survive [26]. Here we confirm embryonic lethality of mice with homozygous deletion of the conditional targeted DIDO3-exon16, and differentiation defects in their ESC. We crossed DIDO mutant mice with the transgene OKSM-4F mice [52] and used reprogrammable OKSM-MEF from WT, DIDOΔNT, and DIDO3ΔE16 mutants. At difference from WT and DIDOΔNT MEF, which reprogram with comparable efficiency, DIDO3ΔE16 MEF were reprogramming-incompetent.

Whole transcriptome analysis of mutant ESC revealed two principal defects; RNA alternative splicing (as in e.g., *DIDO1*, *KLF3*, and *JAM2*) and RNA termination defects (e.g., *WDR33*, *CARM1*, and *POU5f1*). For CARM1, which methylates several splicing factors and enhances exon skipping [53], protein expression was decreased. *POU5f1* is of particular interest, given its role in stem cell maintenance and differentiation [39, 54]. We observed that aberrantly extended 3′UTR resulted in a protein with impaired downregulation at the onset of differentiation.

Our data show that although changes in the 3′UTRs do not alter protein structure, 3′UTR have pleiotropic regulatory functions [55]. Inclusion of ChIP-seq data identified DIDO3 binding sites at the 3′ gene regions. These DIDO sites coincide with RNA pol II S2p, whose binding at 3′ gene ends is implicated in RNA termination by interaction with complexes involved in splicing, cleavage, and polyadenylation [56] and with R-loops, which can pause RNA pol II at termination sites [12, 56–58]. Combined analyses showed that all convergent genes and nearly half of co-orientated genes had RNA anomalies, suggesting a crucial role for DIDO3 on R-loops at gene-rich regions in the genome. These results might define a mechanism by which DIDO regulates R-loops, whose presence impedes transcriptional read-through into neighboring genes [47]. Our data adjust well with the plant BORDER (BDR) protein family, whose domain organization resembles that of DIDO. They act similarly as insulator proteins, preventing transcriptional interference into closely located genes; BDR3 shows a genome binding profile especially similar to DIDO3, with high occupancy at TTS [59].

Regulation of R-loop levels is critical, and misregulation causes DNA damage [13, 21, 60], blocks replication forks, and leads to replication stress [12, 15, 20]. DNA damage is observed in both N- and C-terminal deletion DIDO mutants [24, 25, 27]. In contrast to DIDOΔNT cells, both DIDO3ΔE16 ESC and MEF showed increased R-loop formation.

We found DHX9 association to DIDO3 protein via its DIDO3-specific exon16-encoded domain, and both co-localize with R-loops. The DHX9 helicase is involved in transcription termination [48], and is implicated in physiological as well as pathological R-loop formation, especially when splicing alterations are present [61]. Although it remains unclear whether RNA termination defects in DIDO3ΔE16 ESC are due to impaired unwinding of DNA/RNA hybrids or of dsRNA, we confirmed that DHX9 is needed for correct transcription termination. Since additional DHX9 functions are described [62], including in DNA repair [63], a process implicated in somatic cell reprogramming [64], we cannot exclude that dysregulation of some other functions contribute to reduced reprogramming efficiency in DIDO3ΔE16 MEF.

The results of RNaseH1 overexpression in vivo on DIDO3-dependent termination read-throughs were explainable, as it is exactly the difference between mapping R-loops with the S9.6 mAb or by enzymatically inactive RNaseH1 [65]. RNaseH1 barely maps to TTS, and the existence of RNaseH1-resistant R-loops has been discussed [66]; in vivo, other enzymes very likely play a role. RNaseH2 could be a candidate, and topoisomerase 1 was recently linked to replication stress at R-loop-enriched TTS [67].

Immortalized DIDOΔNT and DIDO3ΔE16 MEF have splicing alterations based on altered SFPQ association [29]. Although these splicing defects are known to increase R-loops [61], the DIDOΔNT mutation with intact DIDO3-exon16 showed no increase in R-loop levels and maintained R-loop regulation capacity. In DIDO3ΔE16, where SFPQ and DHX9 binding are affected, both could nonetheless contribute to elevated R-loop levels.

High R-loop levels can disturb replication progression [13, 15, 19, 60] and lead to replication stress as defined by 53BP1 expression [51]. We detected stress in DIDO3ΔE16 ESC and MEF, and showed that replication stress ultimately impairs DNA synthesis in DIDO3ΔE16 ESC. We detected replication stress exclusively in DIDO3ΔE16 mutants, but neither in DIDOΔNT nor in WT cells, which illustrates the importance of DIDO3 in regulating RNA metabolism.

MEF immortalization overcomes cell damage checkpoints; in DIDO3ΔE16 MEF, replicative stress could be a form of cell damage that contributes to their decreased reprogramming capacity, and E6/E7 immortalization helps restore it.

The importance of R-loop biology has grown in recent years; new insights, their complexity, and available tools have been discussed [68]. Questions remain regarding the DIDO3ΔE16 phenotype, including the players involved and the precise mechanism of R-loop involvement. Our results nonetheless allow us to postulate a central role for DIDO3 in regulating RNA metabolism, and that the DIDO3-specific exon16 is essential for this function. Its deletion triggers defects in RNA splicing and termination, and impedes dissolution of the increased R-loops, both in association with impaired DHX9 helicase recruitment through the DIDO3-exon16-encoded protein domain. Cells suffer genomic instability, DNA damage, and replication stress; ESC thus fails to undergo differentiation and MEF to undergo somatic cell reprogramming.

## Materials and methods

### Construct of a conditional allele with floxed exon16 of the Dido gene

Heterozygous mice with DIDO3-exon16 flanked by LoxP sites were generated by Ozgene Pty Ltd (Bentley, AU). In brief, a LoxP site was inserted at the 3′ end of DIDO intron 15. A neomycin selection cassette (neo, encoding amino 3’-glycosyl phosphotransferase) was inserted downstream of DIDO3-exon16, flanked with flipase recombination target (FRT) sites and followed by another LoxP site and a sequence reproducing the splice acceptor of DIDO3-exon16.

The linearized targeting vector was electroporated into C57BL/6-derived Bruce4 ES cells. Successful homologous recombination was identified by Southern blot screening of antibiotic-resistant ESC colonies. After blastocyst microinjection, chimeric offspring were crossed to C57BL/6 J mice to yield heterozygous *DIDO-tm3Cmar* mice, which were transferred to our animal facility. For Cre recombination we used *Tg(SOX2-Cre)1Amc* mice. To obtain reprogrammable MEF, we crossed these mice further to *Tg(tetO-POU5f1,SOX2,KLF4,MYC)1Srn* mice.

### Cell culture

ESC were cultured in KO-DMEM (Gibco, ThermoFisher) supplemented with 20% fetal calf serum, GlutaMAX (Gibco, ThermoFisher), non-essential amino acids, 2-mercaptoethanol, antibiotics, and murine leukemia inhibitory factor (LIF, Millipore) on a layer of mitomycin C-treated mouse embryonic fibroblasts (MEF) or gelatinized tissue culture plates. MEF were cultured in the same medium without LIF supplement.

For stable transfectants, we used Lipofectamine 2000 (ThermoFisher) according the manufacturer’s protocols; selection was performed with puromycin. To induce differentiation, ESC were cultured in MEF medium without LIF in low-adhesion plates.

All cell cultures were routinely tested for the absence of mycoplasma contamination.

### In vitro reprogramming of primary 4F-MEF

For in vitro reprogramming of primary OKSM-inducible MEF, day E13.5 cells were seeded at a density of 5.5 × 10^5^ cells/cm^2^ in MEF medium in 0.1% gelatin-coated 6-well plates. After 24 h, medium was changed to iPS medium (the same as ESC media with doxycycline (1 μg/mL), Sigma). In the case of the *DIDO3ΔE16* heterozygous mutant and wt MEF, both were infected with Ad5CMV-Cre virus (Viral Vector Core Facility, University of Iowa, Iowa City, IA) at a multiplicity of infection (MOI) of 500, one day before starting doxycycline treatment.

Medium was changed every other day until cells were used for alkaline phosphatase (AP) assays (10–14 days after initiation of doxycycline treatment) or colonies were picked (13–15 days after initiation of doxycycline treatment) and cultured for further characterization.

For in vitro reprogramming of E6/E7 immortalized wt and DIDO3ΔE16 MEF [29], cells were infected with the mixture of the four retroviruses bearing the plasmids encoding OKSM, or with GFP, and cultured in normal ESC medium until day 10, when AP staining was carried out.

We monitored cells for alkaline phosphatase (AP staining kit AB-0300; Sigma) as a first sign of pluripotency. Images of plate wells were analyzed with ImageJ software (NIH) by Analyse Particles (note that regions of interest (ROI) were distinct in experiments with primary or immortalized MEF).

### Cell cycle analysis and EdU incorporation

ESC were labeled with 10 μM EdU (5-ethynyl-2’deoxyuridine; 30 min), processed with the Click-iT Plus EdU Flow Cytometry Assay Kit (ThermoFisher) following the manufacturer’s protocols, and then analyzed in a Beckman Coulter Flow Cytometer FC500. To enrich ESC in G1-phase, cells were cultured (24 h) in ESC medium with 1% FCS and 20 ng/ml bFGF (Invitrogen), and serum-released in normal ESC medium with 20% FCS; 10 μM EdU was then added for 30 min.

### Knockdown and overexpression experiments

Small interfering (si) RNA experiments were performed with ON-TARGETplus SMARTpool specific for mouse DHX9 or, as negative control, with Non-targeting Pool (both from Dharmacon). Plasmids for mouse RNaseH1 overexpression, pCAGG-HARNaseH1-IRES-hygromycin, and Kozak-optimized plasmid were a generous gift from Dr. Thomas G. Fazzio [16]. In both cases, cells were transfected with Lipofectamine 2000 following the manufacturer’s instructions.

### Protein analysis

For immunofluorescence analyses (stemness and differentiation), cells were fixed in 4% paraformaldehyde, permeabilized, and blocked with 0.5% Triton X-100, 6% horse serum in TBS, stained with primary (overnight, 4 °C) and secondary antibodies (2 h, RT), and mounted in DAPI-containing medium.

For immunofluorescence with S9.6, γH2Ax, and 53BP1 antibodies, cells were fixed in ice-cold methanol, permeabilized with 0.1% Triton X-100, and blocked in 5% BSA/PBST. Negative control cells were treated with RNAseH (NEB) at 37 °C overnight before blocking.

For confocal microscopy, we used a Zeiss laser scanning or an Olympus confocal microscope. Images were processed and quantified with ImageJ. Integrated density was calculated above defined thresholds, and the value for negative cells was set at less than the lowest measured value.

For flow cytometry analysis, cells were disaggregated with enzyme-free cell dissociation buffer (Invitrogen) and the surface was labeled with anti-Jam2 rat monoclonal antibody (R&D Systems).

For co- and immunoprecipitation (IP) experiments, cell lysates were prepared in NETN buffer with protease inhibitors (Roche) and 1 mM PMSF, incubated with specific antibodies (4 °C, overnight), followed by Protein A- or G1-loaded magnetic beads (Dynabeads, ThermoFisher; 4 °C, 2 h). Immunoprecipitates were washed six times, released from beads in sample buffer, and used for western blot.

For western blot, cell pellets were lysed in cell lysis buffer (Cell Signaling), 20–40 μg protein solution was loaded for SDS-PAGE, then transferred to nitrocellulose membrane (Bio-Rad), and probed with the indicated antibodies.

Antibodies used are listed below.

### RNA and RT-PCR

Total RNA was prepared with a SPLIT RNA extraction kit (Lexogen, Austria) according to the manufacturer’s protocol, using ~2.5 × 10^6^ cells. RT-PCR amplifications were performed with a Verso One-step RT-PCR Kit (ThermoFisher), using 0.1 μg RNA and specific primers for the amplified genes. Oligonucleotides are listed below.

### DRIP, slot blot, and qPCR

For R-loop precipitation on chromatin, nuclear pellets were lysed in RIPA buffer containing 10% glycerol, protease inhibitor cocktail (Roche), and 1 mM PMSF, sonicated 10 times (10 s on, 30 s off) in a Bioruptor (Diagenode), and incubated with S9.6 mAb coupled to Protein A magnetic beads (Dynabeads; 4 °C, overnight) in the presence of RNaseA (10 ng/ml), followed by SDS-PAGE and western blot as described above for IP.

For R-loop precipitation on DNA, we mainly followed the initial protocol of Ginno et al. [69] as further detailed by Sanz et al. [70], with some modifications. In brief, cell nuclear extracts were prepared and digested with Proteinase K (56 ^o^C, overnight), purified by phenol/chloroform treatment, precipitated with ethanol and resuspended in TE buffer. DNA was digested overnight in the restriction enzyme cocktail, again purified, precipitated, and resuspended in TE buffer. Half the DNA was treated with RNaseH1 (NEB); input samples of both purified with PCR purification columns (Qiagen) were stored; DNA probes, treated and untreated, were diluted in binding buffer and precipitated overnight with S9.6 antibody coupled to Protein A-loaded magnetic Dynabeads. After washing with binding buffer, immunoprecipitates were eluted in TE buffer with SDS, digested with Proteinase K, and purified with the PCR purification kit (Qiagen).

Inputs and precipitates were validated in duplicate by slot blot analysis on positively charged Hybond nylon membrane (Amersham). One half was analyzed on UV-crosslinked membrane for DNA:RNA hybrids with S9.6; the other half was denatured with 0.5 M NaOH, 1.5 M NaCl, and neutralized with 1 M NaCl, 0.5 M Tris-HCl pH 7.5 before UV crosslinking, then probed with mouse single-strand DNA antibody (Millipore) to detect total DNA. Bands of inputs and eluates were quantified using ImageJ.

Inputs and eluates were analyzed for enrichment of specific genes by quantitative PCR with SYBR Green in ABI PRISM7900HT PCR equipment (Applied Biosystems); the percentage of relative DRIP signal was calculated as a ratio of IP/input normalized to standard curves. Oligonucleotides are listed below.

### RNA sequencing

Triplicate RNA probes from WT, DIDOΔE16, and DIDOΔCT+HADIDO3 mESC were sequenced at the Centro Nacional de Análisis Genómico (CNAG-CRG), Barcelona, Spain. Illumina FastQ files containing >77 million paired-end reads per sample were provided by the CNAG-CRG, who also carried out quality control of the FastQ files.

### Computational analysis

Data were obtained, processed, and annotated using R (R Development Core Team, 2014) and Bioconductor programs [71]. A genomic annotation for the UCSC mouse genome build GRCm38/mm10 (file knownGene.txt.gz) was downloaded from the site (ftp://hgdownload.cse.ucsc.edu/goldenPath/mm10/database/). Data for 3′UTR regions were added with the UCSC genePredToGtf tool. To assess read coverage distribution across the genome, bigWig files (10-bp genomic bins) were generated with bamCoverage/deepTools v2.3.1 [72] and normalized for differences. The aligned sequence reads, coverage, and ChIP-seq peaks were visualized with IGVtools [73].

### ChIP-seq analysis

The genomic coordinates for HADIDO3 (GEO: GSE85029), RNA Pol II S2P (GEO: GSE34520), R-loops (GEO: GSE70189) and H3K4me3 (GEO: GSE36114) binding were transformed to the UCSC mouse genome build (mm10) using the liftOver tool (http://genome.ucsc.edu/cgi-bin/hgLiftOver). BED peak files were imported to RStudio and annotated using the R/Bioconductor package ChIPseeker [32]. The promoter region was set to −1 kb to 200 bp of the TSS. We also used the Bioconductor packages org.Mm.eg.db and TxDb.Mmusculus.UCSC.mm10.knownGene for peak annotations. Significance of overlap between ChIP-seq data sets was calculated using the enrichPeakOverlap function implemented in ChIPseeker, setting the number of random permutations (nShuffle) of the genomic locations to 10,000.

### RNA-seq analysis

Sequencing adapters and low-quality reads were removed. The sequences obtained were aligned with TopHat2 software [74]. For read counts and FPKM gene expression estimation, we used Cufflinks version 2.2.1 [75]. We also used DEXSeq [31] to detect differential exon expression. Genes were filtered for significant differential expression using an adjusted P value cutoff at 0.01 (after Benjamini–Hochberg multiple testing correction) and an absolute value of log2 fold change ≥ 0.7 between DIDO3ΔE16 mutant and WT ESC.

To study alterations in 3′RNA isoform abundance, we reanalyzed the RNA-seq data. The Burrows-Wheeler aligner BWA-MEM 0.7.15 (http://bio-bwa.sourceforge.net) was used to align paired-end reads to the UCSC mouse genome build (mm10, http://genome.ucsc.edu/) with standard settings. Alignments were converted to BAM format and de-duplicated with Picard tools 2.9.0 (http://broadinstitute.github.io/picard/). To quantify relative expression at 3′UTR + 2 kb of transcripts, we ran StringTie 1.3.3 [76] using a modified GTF file with mm10–3′UTR annotations, and calculated the transcripts per million (TPM) reads. Sample scaling and statistical analyses were performed with the R package edgeR [73]. Transcripts with TPM > 0 in all samples were kept for downstream analysis. Differentially expressed genes with an absolute value of log2 fold change ≥0.7 and a false-discovery rate (FDR) < 0.05 were considered statistically significant. RNA-seq data have been deposited in the NCBI Gene Expression Omnibus (GEO) under accession number GSE152346.

### Quantification and statistical analysis

The morphology and genotypes of mouse embryos were determined by investigators blinded to experimental groups. For biochemical analyses, a sufficient number of samples per genotype was used to determine biologically meaningful differences between experimental groups based on the variation in parameters as determined in previous studies. For assessment of image data, fields of view were selected to encompass a sufficiently large number of cells to ensure the capture of biologically meaningful differences, guided by previous data. Most results shown are representative of experiments repeated at least three times. Statistical data in graphs are reported as mean ± Standard Error of the Mean (SEM) unless otherwise stated. Experimental group means were compared by unpaired two-tailed Student’s *t* test or ANOVA, depending on the number of groups. Normality of variables was assumed in most analysis, based on published results; variances were similar between experimental groups within each experiment. *P* values < 0.05 were considered significant.

**List of antibodies used**

**Table:** 

Specificity	Antibody	Dilution	Source, reference	Technique
DIDO3	Rabbit polyclonal	1:400	Our laboratory, PAB-Dido3	IF
NT-DIDO	Mouse monoclonal	1:100	Our laboratory, Dido MAB-1C6	IF, WB
OCT4	Mouse monoclonal	1:50/1:1000	Santa Cruz; sc-5279	IF/WB
SOX2	Rabbit polyclonal	1:100	Thermo Scientific; PA1-16968	IF
NANOG	Goat polyclonal	1:25	R&D Systems; AF2729	IF
SSEA1	Mouse monoclonal	1:3	University of Iowa; MC-480	IF
TUJI	Mouse monoclonal	1:500	Covance; MMS-435P	IF
FOXA2	Goat polyclonal	1:50	R&D Systems; AF2400	IF
ASMA	Mouse monoclonal	1:400	Sigma; A5228	IF
AFP	Rabbit polyclonal	1:400	Dako; A0008	IF
KLF3	Rabbit polyclonal	1:800	Sigma-Aldrich; AV32186	WB
JAM2	Rat monoclonal	1:100	R&D Systems; MAB988	IF
CARM1/ PRMT4	Rabbit polyclonal	1:1000	Cell Signaling; 4438	WB
HA-tag	Mouse monoclonal	1:500/1:1000	Covance; MMS-101P	IF/WB
WDR33	Mouse monoclonal	1:100	Santa Cruz; sc-374466	WB
β-Actin	Mouse monoclonal	1:3000	Sigma-Aldrich; A3853	WB
anti-DNA/RNA hybrid, clone S9.6	Mouse monoclonal	1:500/1:1000/10 μg	Merck Millipore; MABE 1095 and purified AB, a generous gift from A. Aguilera	IF/Slot blot/IP
γH2Ax	Rabbit polyclonal	1:600	Bethyl; IHC-00059 Novus Biologicals; NB100-384	IF
DHX9	Rabbit polyclonal	1:1000	Abcam; ab26271	IP,WB
53BP1	Rabbit polyclonal	1:500	Abcam; ab36823	IF,WB
Anti-ssDNA	Mouse monoclonal	1: 4000	Millipore; MAB3868	Slot blot
pCHK1 (Ser345)	Rabbit polyclonal	1:1000	Cell Signaling; 2341	WB

*IF* immunofluorescence, *WB* Western blot, *IP* immunoprecipitation

**Oligonucleotides**

**Table:** 

gene	Forward	Reverse	Method
DIDO WT	GTGTGCTGGCACATTCAGGG	GTATTATATTTGGACGTGGTGATT	Genotyping
DIDO3-LoxP-exon16	GTGTGCTGGCACATTCAGGG	GTATTATATTTGGACGTGGTGATT	Genotyping
DIDO3ΔE16	GTGTGCTGGCACATTCAGGG	TCACATTGCCAAAAGACGGC	Genotyping
KLF3 short isoform	GTATACCAGCCACCTGCAGC	AAACACCAGGTCTGCCTAAG	RT-PCR
KlLF3 long isoform	ACACTAAGAGCTCGCACTTG	CATGGAGAAACCGACAAATTG	RT-PCR
WDR33 short 3′UTR	GTGTCCTGAGAAATGGAGCACC	TCCTACCTTTCCTACCTTTGC	RT-PCR
WDR33 long 3′UTR	AGACGAAAGACTGATGCCGAC	TCCTACCTTTCCTACCTTTGC	RT-PCR
JAM2 short isoform	CCTTGGTCTACTACCAACAGG	GTAATACCATCCAGGCTGCTG	RT-PCR
JAM2 long isoform	CTAAAGTCACTACGATGAGCG	GTCAGACACAAGATGCCAGG	RT-PCR
POU5f1 short 3′UTR	CACATCGCCAATCAGCTTGG	TTGCCTTGGCTCACAGCATC	RT-PCR
POU5f1 long 3′UTR	CACATCGCCAATCAGCTTGG	TCAGCAGTTAGGAGCTATGGC	RT-PCR
HA-primer	GTACCCTTATGACGTGCCCG		RT-PCR
POU5f1	CTGCCCAACTCCATGCTTGG	ATGGCATCGGCTAAAGCACC	ChIP, DRIP
CARM1	GAGGAGGGTACATCCTCAAC	CGGCACTATTGTCTACTGTGG	DRIP
SOD3	GCTTCGACCTAGCAGACAGG	CACCACGAAGTTGCCAAAGT	DRIP

## Supplementary information

Supplemental Information

Suppl. Figure 1

Suppl. Figure 2

Suppl. Figure 3

Suppl. Figure 4

Suppl. Figure 5

Suppl.Figure 6

Suppl. Table 1

Suppl. Table 2

Suppl. Table 3
