# Supplementary material for: Impaired stem cell differentiation and somatic cell reprogramming in DIDO3 mutants with altered RNA processing and increased R-loop levels
Source: Cell Death Dis. 2021 Jun 21;12(7):637. doi: 10.1038/s41419-021-03906-2 (PMC8217545; doi:10.1038/s41419-021-03906-2)
Supplement: Supplementary file 1 — Supplemental Information [file 41419_2021_3906_MOESM1_ESM.docx]

**Supplemental Information**

**Supplemental Figure Legends**

**Suppl. Figure 1. *Analysis of RNA-seq and ChIP-seq data* related to Figure 2**

**a)** Log 2 ratios of exons with a 1.5-fold change in DIDO3ΔE16 versus WT (blue dots) and the same exons recovered in DIDO3ΔCT+HADIDO3 versus WT (red dots); analyzed with the DEXSeq program.

**b) DIDO1 isoform is absent in DidoΔE16 ESC**

IGV image shows the *DIDO* locus with aligned, normalized RNA-seq reads of triplicates of WT, DIDO3ΔE16, and DIDO3ΔCT+HADIDO3 ESC. Arrows indicate missing DIDO1-specific reads in DIDO3ΔE16, and recovered expression after HADIDO3 overexpression in the DIDO3ΔCT mutant.

**c) DIDO3 peaks are present in genomic regions with high gene density**

Association of DIDO3 peaks from ChIP-seq data (GEO: GSE85029) on the chromosomes. In the Y-axis (peak height) shows the MACS score according to the BED file. Numbers at right include HADIDO3 binding sites and gene density per chromosome in parentheses. Mouse chromosomes have a mean gene density of 10.1 ± 2.3 genes/Mbp (Mayer et al., 2005).

**d) Significantly enriched GO terms (biological processes) identified in the differentially expressed genes**

Intersection (%): number of genes and percentage of genes annotated with a given term. fold enrichment (or enrichment score): geometric mean of all the enrichment P-values of associated terms. The whole mouse genome was used as a background list. P-value: significance of gene-term enrichment. P-value <0.05 suggests that term is significantly more enriched than random chance in the study. FDR: Benjamini-Hochberg false discovery rate multiple testing correction of enrichment P-values. As a reference for comparison, we included the general GO term GO:0009987 "Cellular process" which contains about 78-81% of all genes in the two input lists, DEXSeq and 3’UTR-2kb.

**Suppl. Figure 2*. Example genes with altered RNA expression but no difference in protein expression* related to Figure 2**

**a**) *KLF3* example gene: IGV image shows *KLF3*-locus, aligned and normalized RNA-seq reads of triplicates of WT, DIDO3ΔE16, and DIDO3ΔCT+HADIDO3 ESC, as well as DIDO3 ChIP-seq peaks (red) with overlapping RNA pol II S2p peaks (green) and defined 3’UTR regions (blue). Arrows indicate reads of defined 3’UTRs.

**b**) Different abundance of KLF3 short isoform1 and long isoform2 (indicated in a) confirmed in WT, DIDO3ΔE16 and DIDO3ΔCT+HADIDO3 RNA tested by RT-PCR.

**c**) Western blot analysis of lysates of ESC and embryonic bodies without LIF at d1 and d3, comparing KLF3 protein levels in WT, DIDO3ΔE16 and DIDO3ΔCT+HADIDO3 cells.

**d**) *WDR33* example gene: IGV image illustrates *WDR33* locus, aligned and normalized RNA-seq reads of triplicates of WT, DIDO3ΔE16, and DIDO3ΔCT+HADIDO3 ESC as well as DIDO3 ChIP-seq peaks (red) with overlapping RNA pol II S2p peaks (green) and defined 3’UTR regions (blue). Arrow indicates read-through of the last defined 3’UTR.

**e**) Different abundance of WDR33 short 3’UTR and long 3’UTR (indicated in a) confirmed in WT, DIDO3ΔE16 and DIDO3ΔCT+HADIDO3 RNA tested by RT-PCR.

**f**) Western blot analysis of lysates from ESC and embryonic bodies without LIF at d2, comparing WDR33 protein levels in WT, DIDO3ΔE16, and DIDO3ΔCT+HADIDO3 cells.

**Suppl. Figure 3. *Coincidence of R-loops* related to Figure 4**

**a)** DIDO3 binding sites on DNA, alone or with overlapping binding sites of RNA pol II S2p (R2S) and/or R-loops, preferentially at 3’UTR of genes, and their percentage of alterations depending on proximity to neighbor genes.

**b**) Slotblot analysis of DIDO3ΔE16 and WT genomic DNA, untreated or pretreated with different RNase enzymes (H1: DNA/RNA hybrids, III: dsRNA, T1: ssRNA) as specificity control for S9.6 mAb.

**c)** Results of qPCR from DRIP (DNA-RNA immunoprecipitation) inputs and precipitates with S9.6 mAb, alone or RNaseH1-treated; relative DRIP signal as the ratio between IP/input is shown for *CARM1*, *POU5f1*, and *SOD3*.

**d**) Western blot analysis of lysates of WT and DIDO3ΔE16 ESC, both transfected with empty vector=V, mouse HA-tagged RNaseH1=RH1, or Kozak optimized mouse HA-tagged RNaseH1=KRH1.

**e**) Abundance of *POU5f1* short and long 3’UTR in WT and DIDO3ΔE16 ESC transfected with V, RH1, or KRH1 (see d); β-ACTIN was used as control for equal RNA amounts. A representative experiment is shown (n=4).

**f**) Contour density blot of WT and DIDO3ΔE16 ESC (24-h serum-starved G1-enriched cells, serum-released) after a 30 min pulse with EdU, showing incorporation of EdU and reentry into the cell cycle.

**Suppl. Figure 4. *R-loops, DNA damage, and replication stress in DIDOΔNT MEF* related to Figure 5**

**a)** S9.6 fluorescence intensity to detect R-loops and of γ-H2Ax to detect DNA damage was quantified in the nucleus of WT and DIDOΔNT MEF. At least 100 cells were analyzed from three different experiments. Specificity for S9.6 mAb was controlled after RNase H1 treatment. Scatter dot blot with median is shown. Statistical analysis was performed with the one-way ANOVA t-test, ** *P*≤0.01.

**b)** 53BP1 fluorescence intensity to detect replication stress was quantified in the nucleus of WT versus DIDOΔNT MEF. At least 170 cells were analyzed from three different experiments. Scatter dot blot with median is shown.

**Suppl. Figure 5. *Reprogramming of DIDO3ΔE16/4F-MEF* related to Figure 6**

**a**) Deletion efficiency by genotype PCR for WT and DIDO3ΔE16 MEF before and after AdCre infection.

**b**) Deletion efficiency analyzed in western blot with anti-DIDO antibody in lysates of WT and DIDO3ΔE16 MEF before and after AdCre infection, using TUBULIN as loading control.

**c**) Immunofluorescence for the stemness-related markers NANOG, OCT4, SOX2, and SSEA‑1 in DIDO3ΔE16 iPSC colonies. Bar =50 μm.

**d**) Western blot analysis of lysates from iPSC and embryonic bodies from heterozygous and homozygous DIDO3ΔE16 iPSC at different times after LIF withdrawal, monitoring DIDO3, DIDO3ΔE16, and OCT4 protein levels, using TUBULIN as loading control.

**Suppl. Figure 6. *Reprogramming of DIDOΔNT****/****4F****-****MEF* related to Figure 6**

**a**) Immunofluorescence for the stemness-related markers NANOG, OCT4, SOX2, and SSEA-1 in DIDOΔNT iPSC colonies. Bar = 50 μm.

**b**) Immunofluorescence for the differentiation-related markers for all three germ layers (TUJ1, ectoderm marker; ASMA, mesoderm; FOXA2 and AFP, endoderm) in DIDOΔNT iPSC colonies. Bar = 50 μm.

**Supplemental Tables and Legends**

**Suppl. Table 1*. RNA-seq analysis by DEXSeq.* Related to Figure 2.**

Exon expression alterations were obtained with the DEXSeq program (https://bioconductor.org/packages/DEXSeq/). FDR <0.01

index original: exon order

Ensembl_gene_ID: Ensembl gene identifier

dCT_log2FC: exon usage log2 fold change between DIDO3ΔCT+HADIDO3 mutant and WT ESC

dE16_log2FC: exon usage log2 fold change between DIDO3ΔE16 mutant and WT ESC

gene_symbol: Entrez gene symbol

**Suppl. Table 2. *RNA-seq analysis by edgeR*. Related to Figure 2.**

Gene expression alterations were obtained with the edgeR program (https://bioconductor.org/packages/edgeR/)

Gene names in Bold (column F, "gene_symbol") coincide with the ChIPseeker "flank gene" annotation in Table S3 (column Q, "flank_symbol").

dE16_log2FC: Gene expression changes, log2 fold change between DIDO3ΔE16 mutant and WT ESC

dE16_FDR: adjusted p-values

dCT_log2FC: Gene expression changes, log2 fold change between DIDO3ΔCT+HADIDO3 mutant and WT ESC

dCT_FDR: adjusted p-values

transcript_ID: UCSC transcript identifier

gene_symbol: Entrez gene symbol

exons: annotated transcript exons

**Suppl. Table 3*.HADIDO3 ChIP-seq analysis by ChIPseeker*. Related to Figure2.**

ChIP-seq annotations were obtained with the ChIPseeker program (https://bioconductor.org/packages/ChIPseeker/)

A detailed description about the ChIPseeker commands and their output files is available at https://bioconductor.org/packages/release/bioc/vignettes/ChIPseeker/inst/doc/ChIPseeker.html#peak-annotation

Gene names in Bold (column Q, "flank_symbol") means that expression alterations in their 3'UTR were detected by RNA-seq experiments. These genes are in bold in Table S2 (column F, "gene_symbol").

NA: in columns M to Q means that a flank-gene was not detected in the close neighborhood (+/- 5kb) of the binding site.

NA: in column K means that an Ensembl gene identifier is not asigned to a specific gene symbol.

**Suppl. Table 4. *Peak overlap enrichment analysis*. Related to Figure 4.**

| **qSample ^(a)^** | **tSample ^(b)^** | **qLen ^(c)^** | **tLen ^(d)^** | **N_OL ^(e)^** | **N_OL (%qLen) ^(f)^** | **p-value ^(g)^** | **p-adjust ^(h)^** |
| --- | --- | --- | --- | --- | --- | --- | --- |
| HADIDO3 | RNA pol II S2P | 2888 | 4531 | 1900 | 65.8% | 9.9e-05 | 1.5e-04 |
| HADIDO3 | R-loops | 2888 | 53667 | 1793 | 62.1% | 9.9e-05 | 1.5e-04 |
| HADIDO3 | H3K4me3 | 2888 | 23360 | 108 | 3.7% | 0.13 | 0.17 |

^(a)^ Query ChIP-seq sample, ^(b)^ Target ChIP-seq sample, ^(c)^ Number of query peaks, ^(d)^ Number of target peaks, ^(e)^ Number of overlapped peaks between query and target, ^(f)^ Percentage of overlapped peaks, ^(g)^ calculated p-value by ChIPseeker, ^(h)^ p-value correction (FDR) according to the Benjamini and Hochberg method. The values were obtained using the ChIPseeker command enrichPeakOverlap(queryPeak=file1, targetPeak=file-list, TxDb= TxDb.Mmusculus.UCSC.mm10.knownGene, pAdjustMethod="BH", nShuffle=10000, chainFile=NULL, verbose=FALSE) and a number of randomly permutations in the genomic locations of 10000. The ChIP-seq datasets used were: HADIDO3 (GEO: GSE85029), RNA pol II S2P (GEO: GSE34520), R-loops (GEO: GSE70189), and H3K4me3 (GEO: GSE36114).

**Suppl. Table 5**. ***List of genes associated to RNA splicing, RNA termination, RNA elongation rate, and R-loop dynamics*. Related to Figure 4.**

| ***Gene*** | ***Chr*** ^(a)^ | ***ChIP-seq*** ^(b)^ | ***RNA-seq*** ^(c)^ | ***Function*** ^(d)^ |
| --- | --- | --- | --- | --- |
| ***Primary target gene*** | | | | |
| *DHX16* | Chr17 (+) | MACS_peak_1221  MACS_peak_1220 | log_2_FC = -1.07  FDR = 6.49e-04 | mRNA splicing |
| *HMGA1* | Chr17 (+) | MACS_peak_1174  MACS_peak_1175 | log_2_FC = -1.5  FDR = 2.77e-10 | 3'-end processing of mRNA transcripts; binds A+T rich regions |
| *PABPC1* | Chr15 (-) | MACS_peak_924 | log_2_FC = 0.89  FDR = 0.5 ^(e)^ | Binds the poly(A) tail of mRNA; mRNA splicing; mRNA stability |
| ***Secondary target gene (downstream)*** | | | | |
| *SETd5* | Chr6 (+) | - | log_2_FC = -4.17  FDR = 3.28e-26 | Allows on-time RNA elongation dynamics; regulates neural stem cell proliferation |
| *SUGP1* | Chr8 (+) | - | log_2_FC = -1.07  FDR = 2.46e-02 | mRNA splicing |
| ***Confirmed and putative DIDO3 interactions in large protein complexes*** | | | | |
| *DHX9* | Chr1 (-) | MACS_peak_121  MACS_peak_122  MACS_peak_123 | - | Unwinds R-loops; Binds dsDNA, ssDNA, dsRNA, ssRNA and poly(A)-containing RNA |
| *DHX30* | Chr9 (-) | MACS_peak_2837 | - | RNA-dependent helicase; Nervous system development and differentiation |
| *DDX39b* | Chr17 (+) | MACS_peak_1214 | - | Cotranscriptional and nonscheduled  R-loop removal |
| *SETX* | Chr2 (+) | MACS_peak_1482 | - | mRNA splicing efficiency; Splice site selection; R-loop resolution at G‑rich pause sites located downstream of the poly(A) site |

^(a)^ Gene position is represented by chromosome (Chr) number and strand directions in parenthesis (forward (+) and reverse (-)); ^(b)^ Unique identifiers of ChIP-seq peak according to the HADIDO3 dataset (GEO: GSE85029); ^(c)^ Gene expression changes, log2 fold change (log_2_FC) and adjusted p-values (FDR) between DIDO3ΔE16 and WT ESC, were calculated from RNA-seq data in edgeR. ^(d)^ UniProtKB annotations (www.uniprot.org), ^(e)^ A significant alteration with FDR <0.05 was not detected. Hyphens (-) indicate that ChIP-seq peaks or gene expression changes were not detected.
