# Supplementary figures and images for: Impaired stem cell differentiation and somatic cell reprogramming in DIDO3 mutants with altered RNA processing and increased R-loop levels
Source: Cell Death Dis. 2021 Jun 21;12(7):637. doi: 10.1038/s41419-021-03906-2 (PMC8217545; doi:10.1038/s41419-021-03906-2)

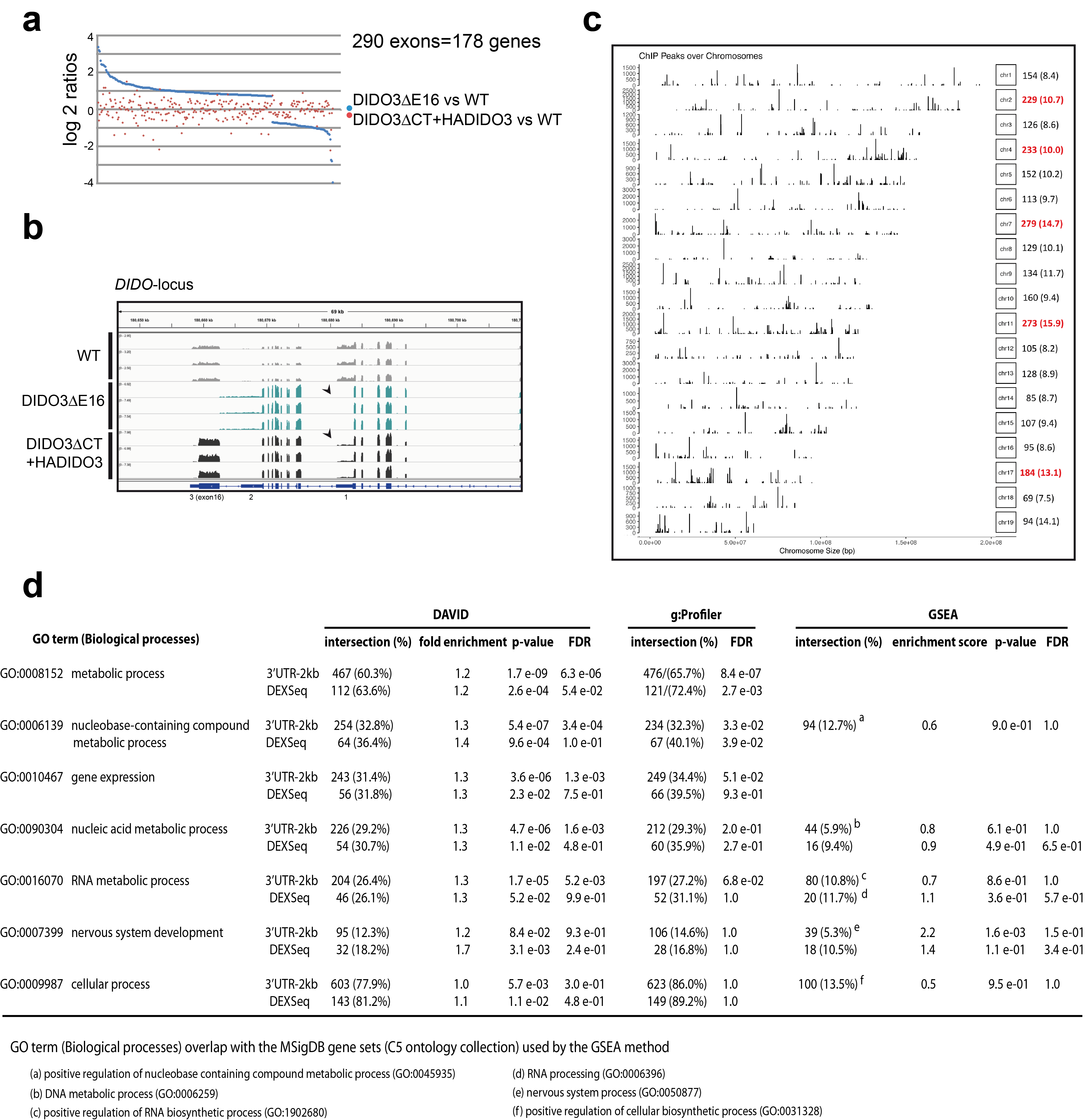

Supplement: Supplementary file 2 — Suppl. Figure 1 [file 41419_2021_3906_MOESM2_ESM.png]

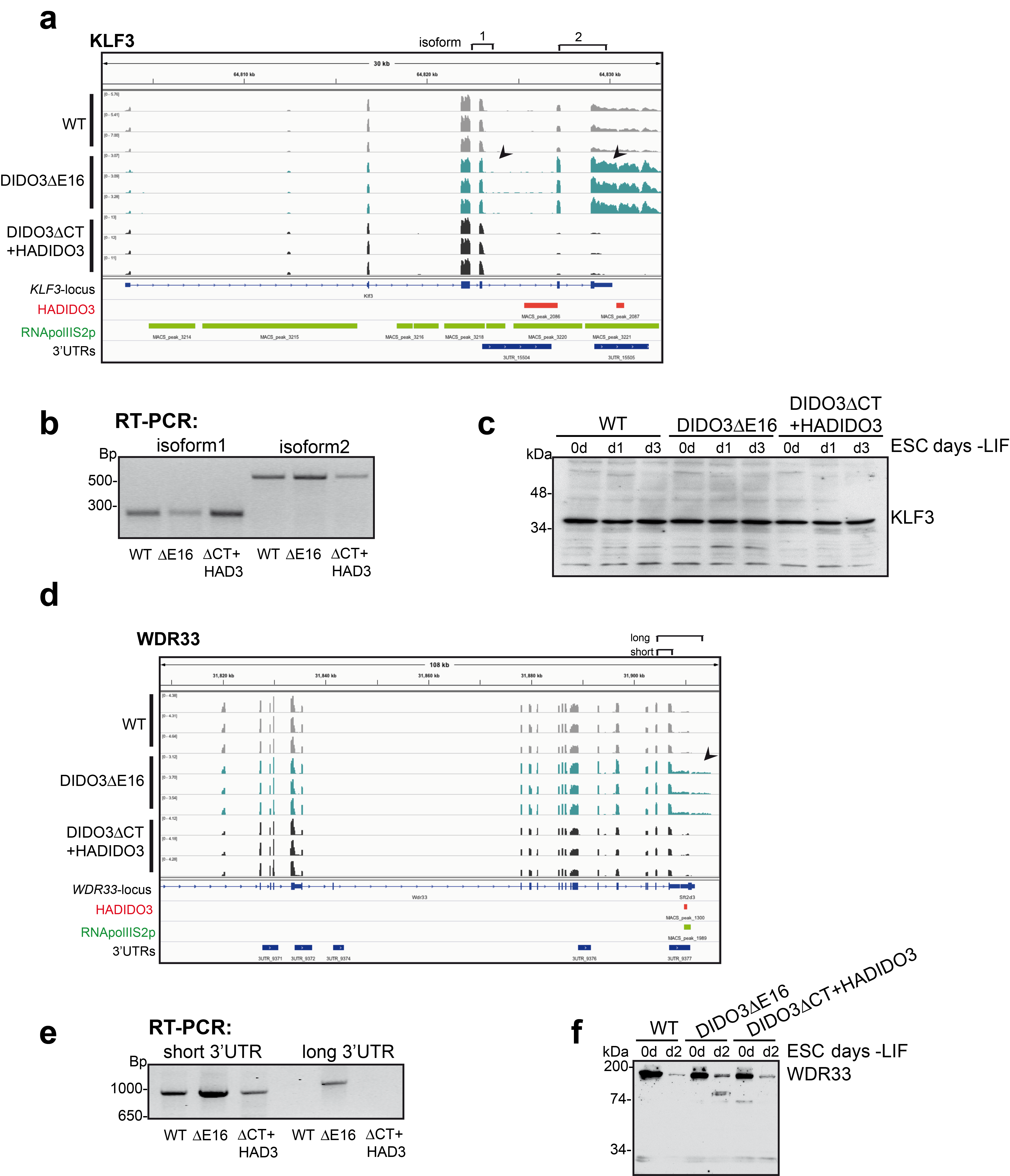

Supplement: Supplementary file 3 — Suppl. Figure 2 [file 41419_2021_3906_MOESM3_ESM.png]

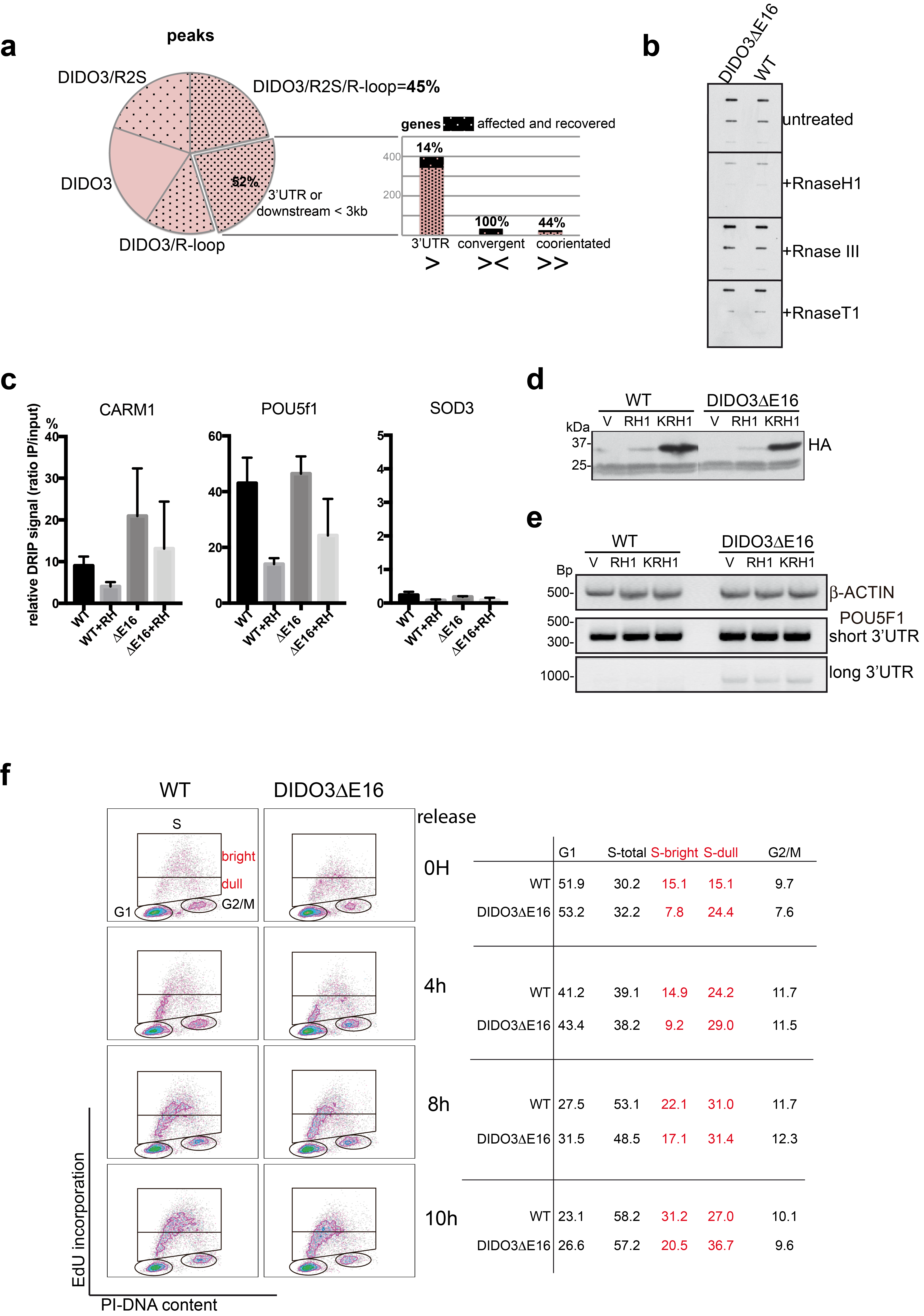

Supplement: Supplementary file 4 — Suppl. Figure 3 [file 41419_2021_3906_MOESM4_ESM.png]

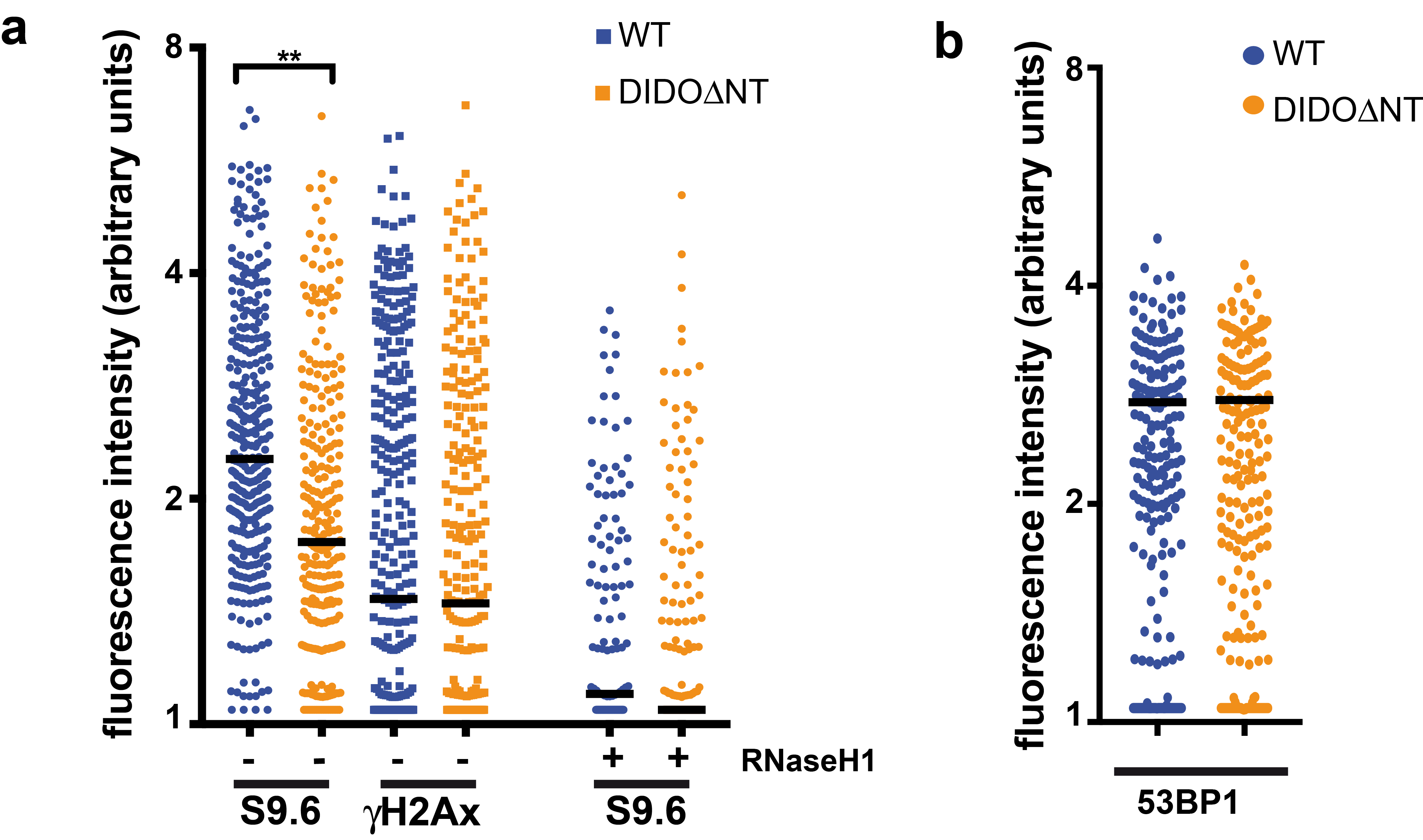

Supplement: Supplementary file 5 — Suppl. Figure 4 [file 41419_2021_3906_MOESM5_ESM.png]

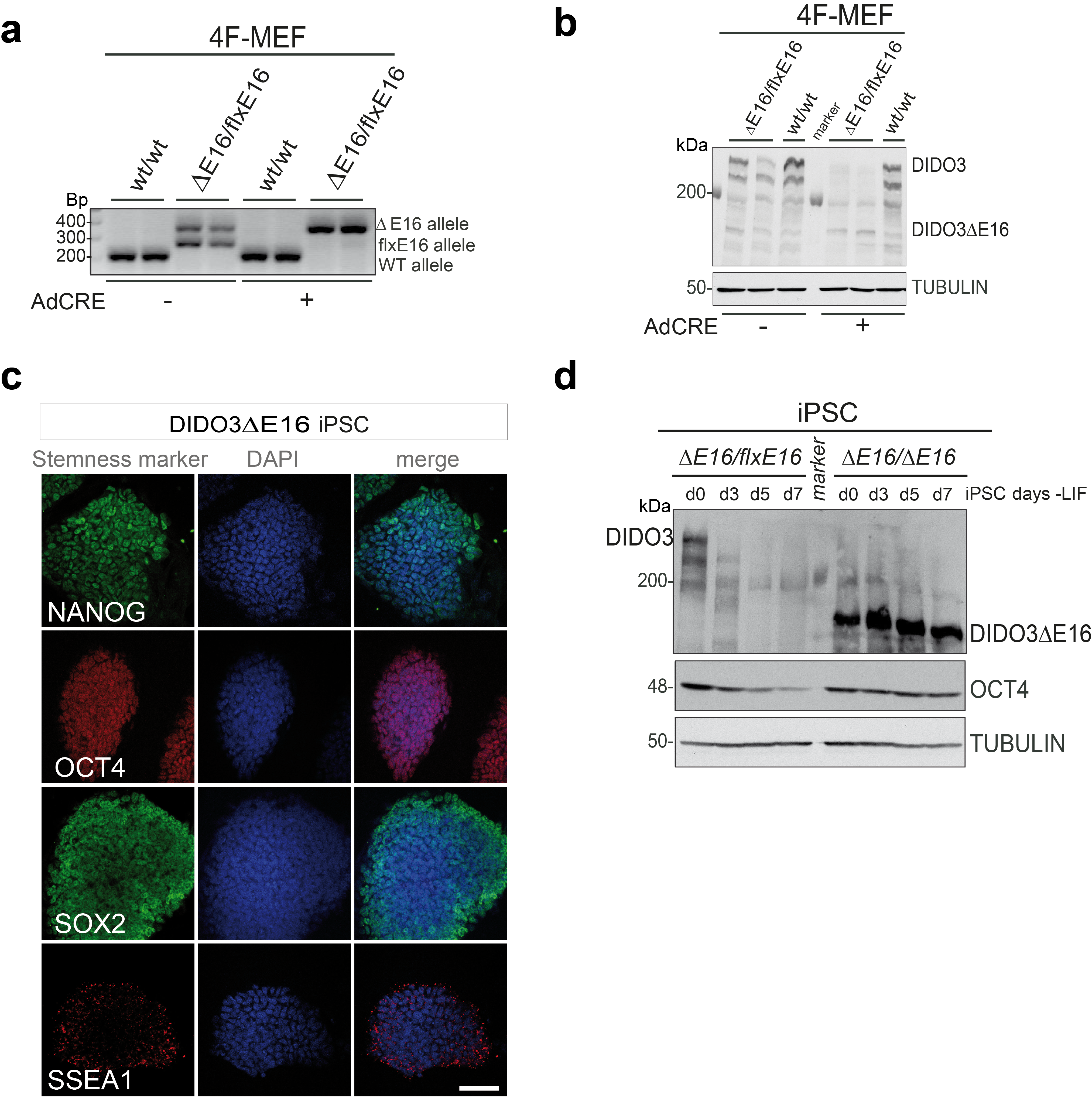

Supplement: Supplementary file 6 — Suppl. Figure 5 [file 41419_2021_3906_MOESM6_ESM.png]

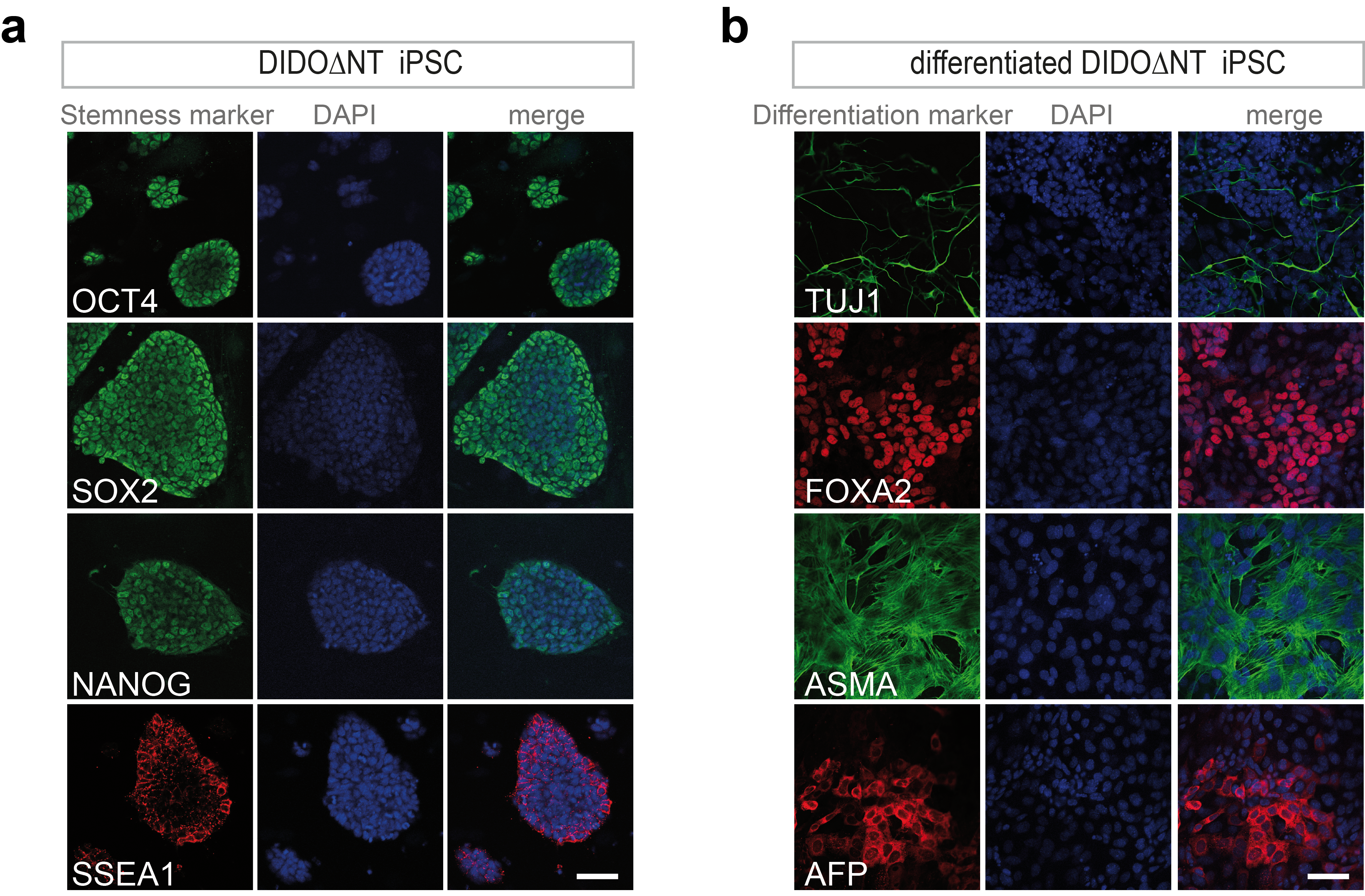

Supplement: Supplementary file 7 — Suppl.Figure 6 [file 41419_2021_3906_MOESM7_ESM.png]
